# Cherokee County Medical Society

**Published:** 1890-10

**Authors:** 


**The Cherokee County Medical Society** held a very interesting meeting July 3rd, at New Birmingham. Dr. B. F. Brittain, President. Puerperal fever was ably and exhaustively discussed, and several valuable papers were read. We regret that we did not receive a report in time to give it in full. This is one of the best and most progressive societies in the State, and they are doing much good work.

